# Neurologist-Led Management of Implantable Loop-Recorders After Embolic Stroke of Undetermined Source

**DOI:** 10.3389/fneur.2021.816511

**Published:** 2022-01-28

**Authors:** Slaven Pikija, Cornelia Rösler, Ursula Leitner, Thomas Zellner, Nele Bubel, Bernhard Ganser, Constantin Hecker, Johannes Sebastian Mutzenbach

**Affiliations:** Department of Neurology, Christian Doppler Medical Center, Paracelsus Medical University, Salzburg, Austria

**Keywords:** embolic stroke of undetermined source, atrial fibrillation, PQ interval, loop recorder, ischemic stroke (IS)

## Abstract

**Introduction:**

Upon completion of the workup for stroke, etiology cannot be identified in approximately one-third of the patients, with an embolic stroke of undetermined source (ESUS) accounting for around 50% of these cryptogenic etiologies. Whether management of complex long-term monitoring in order to detect suspected atrial fibrillation (AFib) could be initiated and managed through a neurologist is not sufficiently investigated.

**Patients and Methods:**

We recruited all consecutive patients with ESUS who received implantation after neurological adjudication of Reveal LINQ^®^ loop recorder between January 2016 and July 2020. We collected demographic, clinical, heart- and neuroimaging, laboratory, and electrocardiographic data assessed on prolonged baseline ECG monitoring, number of supraventricular (SVEs) and ventricular (VEs) extrasystolic complexes, and from preimplantation ECG–PQ interval. AFib detection was manually supervised and determined positive when the duration was over 120 s.

**Results:**

We followed a total of 131 patients for a median of 504 days. There were 45 (34%) manually verified AFib diagnoses. In univariate analysis, earlier implantation after ESUS was associated with AFib detection (13 vs. 31 days, *p* = 0.011). In multivariate analysis, increased rate of AFib was associated with a more prolonged PQ interval (per 50-ms increase) (HR 1.99, 95% CI 1.39–2.85) and number of SVEs (HR 1.29, 95% CI 1.05–1.57) measured on pre-implantation ECG.

**Conclusion:**

We observed similar predictors for Afib after ESUS, albeit with higher frequency than previously reported. This study suggests that the neurologist-led decision, management, and evaluation of ILR after ESUS is feasible.

## Introduction

Stroke continues to be a significant health problem as it was the second leading cause of disability-adjusted life-years worldwide in the year 2019 (1). Around 90% of events responsible for stroke are potentially treatable (2). As a relatively new clinical construct, an embolic stroke of an undetermined source (ESUS) is responsible for half of these cryptogenic strokes (3). This concept was developed to account for strokes of non-lacunar origin in whom embolism, covert atrial fibrillation being the most common source, is the likely stroke mechanism. However, empirical treatment without proven emboligenic heart rhythm cannot be recommended, as shown by the NAVIGATE-ESUS study. Hence, the detection of AFib or atrial flutter is essential before starting oral anticoagulation (4). Therefore, long-term continuous monitoring to detect AFib is recommended following ESUS (5). Timely instituted anticoagulation lowers the incidence of recurrent disabling stroke and its other consequences, such as vascular dementia (6). Implantable loop-recorder (ILR) is considered safe and provides excellent long-term observation due to automatic reporting with little discomfort to the patients. It is superior to long-term wearable monitors in the detection of AFib in non-cardioembolic stroke (7). Previous studies showed AFib detection after long-term observation in up to 30% of patients after cryptogenic stroke (8–11).

Several P-wave indices are associated with AFib detection after ESUS such are PQ interval, and P-terminal force in the precordial lead V1 (PTFV1). It is considered that these indices and especially the prolongation of PQ interval reflect the underlying atrial degenerative process (12, 13). In addition, larger atrial volume, interatrial conduction block, supraventricular premature complexes, extrasystolic complexes, subclinical atrial tachyarrhythmia, the presence of leukoaraiosis, elevated levels of nt-proB-Natriuretic peptide (pro-BNP), age, and CHA_2_DS_2_-VASc score predicted the occurrence of AFib (6, 10, 11, 13–16).

Whether the ILR implantation could be indicated, performed, and managed through a neurologist, is not sufficiently investigated.

## Methods

### Criteria for Implantation

The indication for implantation was made by a neurologist with experience in cerebrovascular diseases (stroke physician). Patients with acute neurological deficits were admitted to the stroke unit of our neurological department and comprehensively evaluated to establish stroke causality. Neuroimaging with magnetic resonance imaging (MRI) or, if contraindicated, with computed tomography (CT) was performed in all patients. CT and MRI angiography and high-resolution color-coded duplex ultrasound scans for vascular imaging and transthoracic echocardiography for heart imaging were performed in all the patients. Each patient was monitored by telemetry on the stroke unit alongside prolonged wearable Holter ECG monitoring (Digitalrecorder EP820, Gepamed, Wien, Austria) with a median duration of 72.0 (IQR 69–72) h. Experienced vascular neurologists (JM, CR) evaluated CT or MRI images for the presence of embolic-type of stroke before implantation of loop recorder device. Upon etiologic evaluation, diagnosis of embolic stroke of undetermined source (ESUS) was established per proposed criteria (3). Other etiologic diagnoses were not eligible for loop implantation. The qualifying ESUS event was classified as stroke or transitory ischemic attack (TIA) with a non-lacunar stroke syndrome lasting <24 h and without evidence of infarction on neuroimaging, both due to ESUS. We have no record regarding specific exclusion criteria.

### Demographics and Clinical Criteria

Modified Rankin Scale (mRS) was assessed on admission as pre-mRS (before qualifying ESUS event) and mRS at admission and rated by a certified examiner (JM, SP, CR) and coded with a number ranging 0–5 (0: no symptoms, 5: severe disability). A certified examiner assessed the National Institutes of Health stroke scale (NIHSS) (ranging from 0 to 42) at admission. Presence (also newly diagnosed) of arterial hypertension, diabetes mellitus, heart failure, prior myocardial infarction, the peripheral arterial occlusive disease was questioned upon or extracted from medical sheets, coded, and recorded in an electronic database. There is a comprehensive collaboration between general practitioners and the hospital system in Austria, so electronic records are readily available. On discharge, intake of antithrombotic medications (aspirin, clopidogrel, ticagrelor, and prasugrel) was recorded. CHA_2_DS_2_-VASc score (congestive heart failure (1 point); hypertension (1 point); age (> 75 = 2 points); diabetes mellitus (1 point); stroke/tia (2 points); vascular disease, e.g.. PAD, previous myocardial infarction, severe calcification of the aorta) (1 point); age: 65–74 (1 point); sex category: women (1 point): range 0–10 points, generally, in the presence of AFIb more than 1 point warrants anticoagulation therapy), calculated independent from AFib detection was evaluated and recorded. Left ventricular ejection fraction was noted and categorized during hospitalization upon heart echocardiography (Logiq S8, GE Ultrasound Korea, Ltd., Korea). The left atrial diameter (LAD) was measured in the parasternal longitudinal axis and grouped into normal (<4.5 cm diameter) and enlarged (≥ 4.5 cm in diameter). Also, the presence of patent foramen ovale (PFO) was noted as a binary variable. Imaging (CT or MRI) was assessed for the presence of (1) embolic-like infarction; (2) Leukoaraiosis grade according to Fazekas ranging from 0 to 3; (3) Site of acute infarction was divided into the middle cerebral artery, anterior cerebral artery, posterior circulation, or multiple locations; (4) Presence of fragmented infarction (as a binary variable) (17). Following laboratory values were extracted from medical records at admission and entered as continuous variables in the database: LDL-C in mg/dl, pro – B Natriuretic peptide (pro-BNP) in pg/ml, Troponin-T in ng/ml, D-Dimer in mg/L. Admission ECG was analyzed for PQ Interval recorded as continuous variables.

The number of supraventricular runs (SV Runs), ventricular extrasystole (VEs), VEs couplets, and/or triplets was detected in wearable Holter-Monitoring during hospitalization and recorded as continuous and as binary variable (noting presence or absence of).

The date of eventual AFib detection as the main endpoint of the study was noted. Also, other significant arrhythmias, when discovered, were entered as type and binary variables. Presence, date, and type of eventual stroke or TIA were recorded.

#### Implantable Loop Recorder and Follow-Up

After the indication for implantation was made, the patients continued to be cared for by the neurological team. Upon establishing ESUS etiology, eligible patients were informed about the procedure, consent would be obtained, and implantation discussed. Upon signing of informed consent, the patient received the loop recorder device. We used Reveal LINQ (RLA) (Medtronic Inc., Minneapolis, MN, USA), with automatic data transmission through the Medtronic CareLink Network, automatic AFib detection algorithm, and remote monitoring. The patients were implanted during hospitalization or have been called upon and implanted in a day-hospital setting. Implantation was performed by a stroke physician in topical anesthesia with a small skin incision in the left parasternal third to fourth intercostal space. The patients were instructed how to use remote systems for daily transmissions. An automatic detection algorithm would send each tachycardia episode, regardless of duration, to manual analysis.

On the other hand, AFib episodes (not tachycardic) would be sent to manual verification when they lasted 120 or more seconds. As proposed previously, a duration of at least 120 s was used to diagnose AFib. The stroke physician would confirm the diagnosis, and in doubt, the cardiology department is consulted. The patient would be called and informed upon when AFib or another significant arrhythmia during the observation period occurred. Explantation of the device was possible throughout the study and performed compulsory upon battery expiration (3 years typically). Data were analyzed at the time of death, voluntary or mandatory (after battery life-cycle expiration), loop recorder explantation, or completion of a minimum of 6 months of follow-up (Supplementary Figure V).

#### Statistical Analysis

Data were expressed as median (interquartile range) due to non-normal distribution and analyzed for the difference between groups (AFib positive and AFib negative) using non-parametrical methods (Fisher Test and Kruskal–Walis test). The significance threshold was set at 0.05. The troponin T and pro-BNP values were log-transformed due to non-normality distribution. Furthermore, the number of SVEs per hour, number of VEs per hour, number of VEs couples, and triplets were transformed using inverse hyperbolic syne (IHS) transformation:


$$\begin{array}{l} \text{f}\left(\text{y},\text{θ}\right)=\text{sinh}-1\left(\text{θy}\right)/\text{θ}=\text{log}\left[\text{θy}+\left(\text{θ}2\text{y}2+1\right)1/2\right]/\text{θ}, \end{array}$$

where θ>0. For any value of θ, zero maps to zero.

Before entering data into multivariate analysis, they were checked for colinearity. As expected, age was correlated with the CHA_2_DS_2_-VASc score since it is a component of the score, so age was not used in further multivariate analysis. Pro-BNP and Troponin T were both correlated to age and all electrographic parameters (IHS-transformed number of SVEs per hour, number of VEs per hour, number of SV runs, number of VEs couples, number of VEs triples) excluding PQ interval per 50 ms increase. Almost all electrographic parameters were correlated, excluding associations with PQ interval per 50 ms increase.

The occurrence of AFib was estimated by the Kaplan–Meier method in the group of patients with and without VEs. A log-rank test was used to account for statistical differences. The PQ interval was analyzed as continuous and per 20 and per 50 ms value increase. Furthermore, PQ interval was categorized according to the presence of first-degree atrioventricular block, i.e., ≤ 200 and >200 ms. Cox regression models were used to assess potential predictors of the rate of AFib detection by the loop recorder during follow-up. We performed several tests with the cox-proportional hazard model. First, we used univariate Cox regression on variables showing *p* < 0.1 in univariate between-group (AF-detection vs. none) analysis. We constructed 3 models for multivariable cox regression analysis. Each was tested for proportional hazard assumptions (cox.zph function in the R survival package). The first model combined clinical variables represented by CHA_2_DS_2_-VASc score and presence of left atrial enlargement (as a binary variable) with electrocardiographic variables (showing *p* < 0.05 association in univariate cox regression analysis): PQ interval per 50 ms increase, and other electrographic parameters showing the least correlation with each other: transformed SVEs per hour and transformed the number of VEs triplets. The second model focused on binary electrocardiographic predictors and included PQ interval per 50 ms increase, SVEs runs present (yes vs. no), and presence of VEs beats (VEs, VEs couplets, and triplets) stratified into a single binary variable (present, non-present). The third model included clinical data (represented through CHA_2_DS_2_-VASc score), laboratory biomarkers, such as log-transformed troponin T and pro-B; electrocardiographic properties, such as PQ interval per 50 ms increase, transformed the number of VEs triplets, and the presence of left atrial enlargement (as a binary variable). The results of the third model did not change when TropT and proBNP were combined into a single variable.

When the PQ interval was substituted with a value per 20 ms increase or as a categorical variable ( ≤ 200 vs. >200 ms), the results did not essentially change. Statistical analysis was performed in R (18).

## Results

From March 2016 to July 2020, 133 patients were recorded in our database. Brain CT was performed in 14 (11%), and MRI was in 116 (89%) of cases. Intracranial CT-angiography was performed in 43% of patients with CT, and intracranial MRI—angiography in 98% of patients with MRI. Left ventricular ejection fraction was categorized echocardiographically to normal (> 55%), mild (45–54%), moderate (30–44%), and severe (<30%). To establish the eventual presence of patent foramen ovale, a transcranial doppler and transcubital bubble test was performed in 82% of patients.

There was one patient with immediate post-implantation pain in the area. Because of the pain, the patient declined to activate the device, so there was no recorded data. Further, one patient was excluded due to an unknown stroke date. This left 131 patients for final analysis. The median age was 71.6 (IQR 61.9–77.4, 54.2% man). Further baseline demographic characteristics are summarized in Table 1.

**Table 1:** Demographic, clinical, heart- and neuroimaging, laboratory, and electrocardiographic data of 131 patients with implantable loop recorder due to embolic stroke of undetermined source in Christian–Doppler–Klinik, Salzburg in the time period 2016–2020.

**Variables**	**Total *N* = 131**	**Atrial fibrilation detected *N* = 45 (34%)**	**Not detected *N* = 86 (66%)**	***P***
Age at implantation (years)	71.9 (62.5–77.4)	75.5 (70.2–78.2)	66.9 (59.9–74.3)	<0.001
Gender (men)	71 (54.2)	25 (55.6)	46 (53.5)	0.855
Stroke/TIA in history	38 (29.0)	20 (44.4)	18 (20.9)	0.008
Nr. of stroke/TIA in history	0.0 (0.0–1.0)	0.0 (0.0–1.0)	0.0 (0.0–0.0)	0.003
Pre-mRS	0.0 (0.0–0.0)	0.0 (0.0–1.0)	0.0 (0.0–0.0)	0.016
Arterial hypertension	98 (74.8)	38 (84.4)	60 (69.8)	0.090
Diabetes melitus	17 (13.0)	6 (13.3)	11 (12.8)	1.000
Myocardial infarction	5 (3.8)	4 (8.9)	1 (1.2)	0.047
Peripheral arterial occlusive disease	4 (3.1)	2 (4.4)	2 (2.3)	0.607
Event to implantation in days	20.0 (8.0–74.5)	13.0 (8.0–26.0)	31.5 (9.0–117.5)	0.011
CHA_2_DS_2_-VASc score				0.051
Median score	4.0 (3.0–5.5)	5.0 (4.0–6.0)	4.0 (3.0–5.0)	0.002
2	14 (10.7)	2 (4.4)	12 (14.0)	
3	21 (16.0)	3 (6.7)	18 (20.9)	
4	31 (23.7)	10 (22.2)	21 (24.4)	
5	32 (24.4)	14 (31.1)	18 (20.9)	
6	28 (21.4)	14 (31.1)	14 (16.3)	
7	4 (3.1)	2 (4.4)	2 (2.3)	
8	1 (0.8)	0 (0.0)	1 (1.2)	
Heart workup				
Left atrium enlargement	35 (26.9)	18 (40.0)	17 (20.0)	0.022
Left ventricular function^*^				0.117
Normal	117 (91.4)	38 (84.4)	79 (94.0)	
Mild impaired	10 (7.7)	6 (13.3)	4 (4.7)	
Moderately impaired	1 (0.8)	0 (0.0)	1 (1.2)	
Neuroimaging				
Fazekas score (0–3)	1.0 (0.0–2.0)	1.0 (1.0–2.0)	1.0 (0.0–2.0)	0.053
Site of infarction^†^				0.762
Anterior cerebral artery	2 (1.5)	1 (2.2)	1 (1.2)	
Middle cerebral artery	96 (73.8)	32 (71.1)	64 (75.3)	
Multiple territories	8 (6.2)	2 (4.5)	6 (7.1)	
Posterior cerebral artery	24 (18.5)	10 (22.2)	14 (16.5)	
Infarction fragmented	9 (6.9)	0 (0.0)	9 (10.6)	0.027
Laboratory values^e^				
LDL-C (mg/dl)	123.0 (88.0–152.0)	112.5 (81.0–146.0)	125.0 (100.0–153.0)	0.155
Troponin-T (ng/ml) (missing 25)	10.9 (7.2–17.1)	16.9 (10.9–21.1)	8.5 (6.5–13.6)	<0.000
Pro-BNP (ng/ml) (missing 16)	168.0 (91.0–402.0)	303.5 (162.2–489.8)	141.0 (69.0–298.0)	<0.000
ECG information				
PQ interval (ms)^£^	160.0 (150.0–188.5)	172.0 (152.0–200.0)	160.0 (148.0–180.0)	0.022
PQ interval >200 ms	12 (9.7)	7 (16.3)	5 (6.2)	0.108
Holter time in hours^¥^	72.0 (69.0–72.0)	72.0 (66.0–72.0)	72.0 (72.0–72.0)	0.153
Ventricular extrasystole (VEs), N^†^^†^	65.0 (4.2–480.0)	250.0 (23.8–1225.0)	40.0 (0.0–200.0)	0.002
VEs pro hour^†^^†^	1.4 (0.1–8.0)	4.5 (0.5–18.1)	0.6 (0.0–3.5)	<0.001
SVEs, N^¥¥^	100.0 (10.0–652.5)	340.0 (20.0–1190.0)	50.0 (0.0–300.0)	0.008
SVEs pro hour^¥¥^	1.4 (0.1–9.3)	5.6 (0.8–16.5)	0.8 (0.0–6.9)	0.004
SV runs, N^†^^†^	0.0 (0.0–2.7)	1.5 (0.0–8.0)	0.0 (0.0–1.0)	0.006
VEs—Couples, N^†^^†^	0.0 (0.0–2.0)	1.0 (0.0–4.0)	0.0 (0.0–1.0)	0.008
VEs—Triplets, N^†^^†^	0.0 (0.0–0.0)	0.0 (0.0–0.0)	0.0 (0.0–0.0)	0.061
Any VEs present^†^^†^	98 (78.4)	39 (88.6)	59 (72.8)	0.044
Follow-up arrhythmy^*^				0.018
AVB G1	3 (2.3)	3 (6.8)	0 (0.0)	
AVB G2	1 (0.8)	1 (2.3)	0 (0.0)	
Sinus arrest	8 (6.2)	4 (9.1)	4 (4.8)	
Follow-up clinical				
Recidivious stroke present	8 (6.2)	1 (2.3)	7 (8.2)	0.263
Death in follow-up	1 (0.8)	1 (2.2)	0 (0.0)	0.349

*Data are number (percentage) or median (interquartile range) when not otherwise specified*.

*Data are given as a number (percentage) or median (interquartile range); N, number; TIA, transient ischemic attack; pre-mRS, modified Rankin scale prior to qualifying ESUS event; LDL-C, low-density lipoprotein; BNP, b-natriuretic peptide; AVB, atrioventricular block; ms, milliseconds*.

* *Unknown in 3 (2.3%) patients (1 in AFib and 2 in non-AFib group). Unknown in 1 (0.8%) patient (non-AFib group). eLDL-C is missing in 1 patient (2.2%) (AFib group); troponin T missing in 6 (13%) AFib group and in 13 (15%) of non-AFIb group patients; pro-BNP missing in 7 (15%) of AFib group and in 9 (10%) of non-AFib group patients. £Missing in 2 (4%) AFib group and in 5 (6%) of non-AFib patients. ¥Missing in 2 (1%) of patients. Missing in 5 (4%) of patients. ¥¥Missing in 7 (8%) of patients*.

Patients received a loop recorder median of 20 days (IQR 8.0–74.5) after a qualifying ESUS event and were followed for a median of 504 days (IQR 142.0–1,166.0).

**Figure 1:**
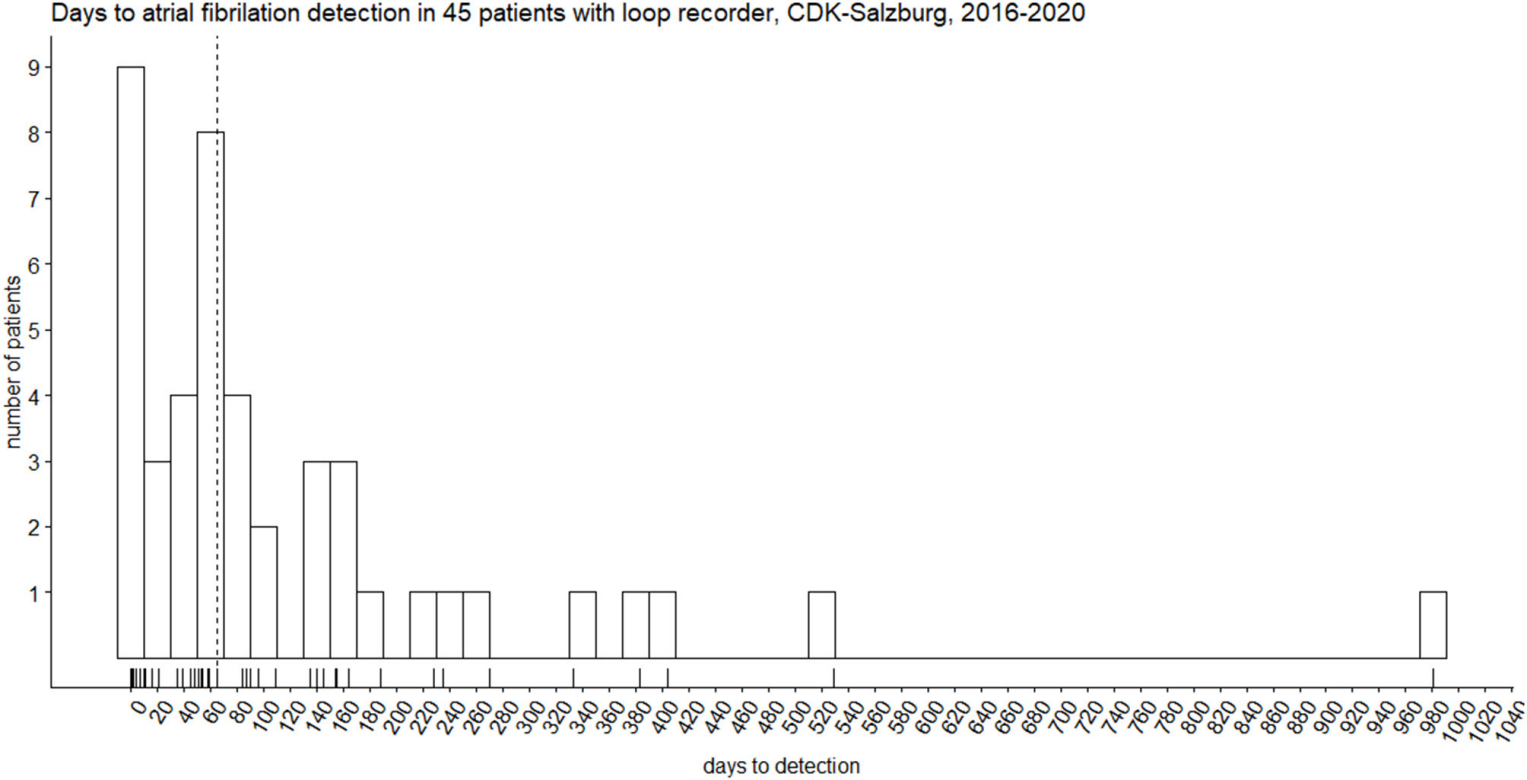
Biphasic distribution of days to atrial fibrillation detection in 45 patients with Reveal LINQ^®^ Medtronic implantable recorder. The vertical dotted line represents the median (65 days).

**Figure 2:**
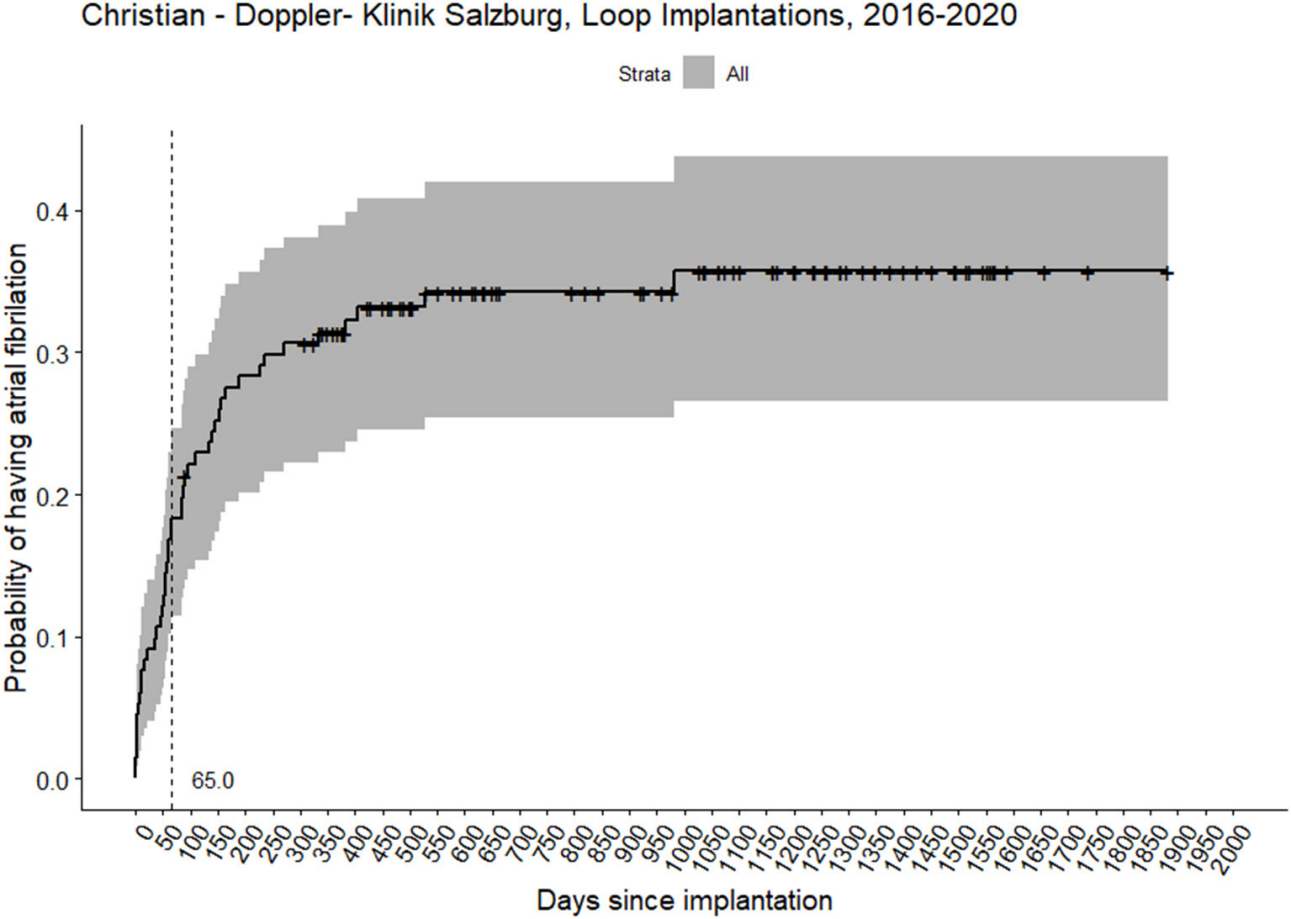
Probability of having atrial fibrillation in 131 patients with the implanted loop recorder device. The vertical dashed line represents median days to atrial fibrillation detection. Crosses represent censored observations.

We discovered forty-five (34%) newly detected AFib that lasted more than 120 s. Median time in days from the initial event (Stroke/TIA) to AFib detection 102.0 days (IQR 57.0–209.0). The median time in days from implantation to AFib detection was 65.0 days (IQR 21.0–154.0) (Figures 1, 2). Two patients (1.5%) had AFib on the same day of implantation; the maximum extended period was 981 days (2.6 years) after implantation.

### Patients' Safety

Apart from one case of post-insertion pain, the devices were well tolerated, and no patients were lost to follow-up.

### Follow-Up

The majority of false-positive automatic AFib detections were due to algorithms oversensing. Recurrent strokes/TIA occurred in 8 (6.2%) of patients, of them in 1 (2.2%) in AFib positive group (n.s.). One patient with AFib had a recurrent stroke after AFib detection, despite treatment with dabigatran etexilate at the time.

### Factors Associated With AFib Occurrence in ESUS Patients

Age was significantly associated with AFib detection (median 75.5 vs. 66.9 years, *p* < 0.001) and comorbidity, as reflected in the CHA_2_DS_2_-VASc score (median 4.0 vs. 3.0, *p* = 0.0021). Previous stroke or TIA in history, prestroke modified Rankin Scale (regarding qualifying ESUS event), and history of myocardial infarction were all statistically worse or more frequent in the AFib group. Implantation performed sooner after the qualifying ESUS event was statistically significantly associated with the later occurrence of AFib (median 13.0 vs. 31.5 days, *p* = 0.011). The site of infarction was not different between AFib groups. However, infarct was significantly more frequently fragmented as per study criteria in the non-AFib group. The presence of left atrial enlargement was statistically more frequent in the AFib group (40 vs. 20%, *p* = 0.022).

### Electrocardiographic Characteristics

The interval of PQ in milliseconds (ms) was significantly longer in the AFib group (median 172 vs. 160 ms, *p* = 0.022). The number of VEs and SVEs was significantly higher in the AFib group (median 250 vs. 14, *p* = 0.002 and 340 vs. 50, *p* = 0.008 in AFib vs. non-AFib group, respectively). Furthermore, the number of SV runs (over three consecutive SV beats), VEs Couplets, and Triplets (each as more > 1 or > 2 ventricular extrasystole after another, respectively) were all significantly associated with AFib (Table 1). Sinus arrest and atrioventricular block grades I and II were diagnosed more often in the AFib group, *p* = 0.018. Correlation testing was performed between various parameters. Results are shown in Supplementary Figure I. Shortly, most of the arrhythmic electrocardiographic parameters (SVEs, VEs) were correlated with each other and with age. However, the PQ interval was not correlated with either of them. Receiver operating curve analysis was performed for VEs, SVEs per hour, and PQ interval with best results for the number of VEs, cut-off 4.0 VEs per hour, area under the curve 0.68 (95% CI 0.59–0.78), sensitivity 54.5%, specificity 78.0% (Supplementary Figures II–IV).

Univariate cox proportional regression analysis revealed the following statistically significant associations with AFib detection: positivel associations were shown for age (per year), CHA_2_DS_2_-VASc score per one-point increase, presence and number of clinical stroke/TIA events in history, pre-mRS, presence of myocardial infarction, presence of left atrial enlargement, mild left ventricular impairment (numbers are but small), log-transformed troponin T and pro-BNP levels, PQ interval in ms and per 50 ms point increase, IHS-transformed SVEs and VEs per hour, IHS-transformed number of SV runs, presence of SV runs (yes vs. no), and IHS-transformed number of VEs-couples and -Triplets number (Supplementary Table I).

In the first multivariate model (see Methods), we found that AFib detection hazard is positively and significantly associated with PQ interval per 50 ms increase and IHS-transformed number of SVEs per hour. When CHA_2_DS_2_-VASc was substituted for age, age was significantly associated with AFib, HR 1.04 (95% CI 1.01–1.08); however, the pseudo-R^2^ was lower. In the second parsimonious multivariate model, we found positive associations with PQ interval per 50 ms increase. In the third model, the significant positive association was seen again for the PQ interval per 50 ms increase (Table 2). The first model performed the best, although it had a mediocre measure of explained variance of 0.215 (maximal 1.0).

**Table 2:** Multivariate cox-proportional hazard analysis of predictors for atrial fibrillation in 131 patients with embolic stroke of undetermined source.

**Variables**	**β coefficient**	**HR (95% CI)**	***P***
**First model**, *N* = 115, Pseudo-R^2^: 0.236, Wald test: 33.2
PQ interval per 50 ms increase	0.68	1.99 (1.39–2.85)	<0.001
CHA_2_DS_2_-VASc score	0.19	1.22 (0.94–1.59)	0.142
SVEs per hour (IHS)	0.25	1.29 (1.05–1.57)	0.015
VEs triplets (IHS)	0.36	1.44 (0.95–2.19)	0.084
Left atrial enlargement	0.51	1.67 (0.86–3.22)	0.127
**Second model**, *N* = 118, Pseudo-R^2^: 0.145, Wald test: 18.7
PQ interval per 50 ms increase	0.70	2.02 (1.38–2.94)	<0.001
SVEs runs present (yes vs. no)	0.54	1.72 (0.91–3.25)	0.096
Any type of VEs present (yes vs. no)	0.94	2.56 (0.99–6.62)	0.053
**Third model**, *N* = 102, Pseudo-*R*^2^: 0.205, Wald test: 28.3
PQ interval per 50 ms increase	0.59	1.82 (1.26–2.64)	0.002
CHA_2_DS_2_-VASc score	0.10	1.10 (0.80–1.53)	0.553
Troponin-T (ng/ml) (log)	0.06	1.06 (0.72–1.56)	0.752
Pro-BNP (ng/ml) (log)	0.19	1.21 (0.89–1.65)	0.219
VEs Triplets (IHS)	0.39	1.49 (0.98–2.50)	0.060
Left atrial enlargement	0.69	2.01 (0.96–4.20)	0.064

*log, log-transformed; HIS, inverse hyperbolic transformation*.

The PQ interval was also tested in 20 ms increase steps and showed in the first model significant positive association with AFib detection [HR 1.09 (95% CI 1.02–1-16), *p*=0.013].

## Discussion

In neurologically-led evaluation and indication for an ILR, AFib was detected in 34% of patients with ESUS. Most events were recorded in the first 2 months after implantation. Lengthening of PQ interval was associated with the two-fold increased rate of AFib in the follow-up period, i.e., each increase of 50 ms in PQ interval was associated with a 67% increase in the expected AFib hazard.

Our detection rate (31.3% at 12 months, 33.6% at 18 months, median time from implantation to detection 65 days) was substantially higher than in CRYSTAL AF Study (12.4% at 12 months and 17.0% at 18 months), in spite of that, we used a more conservative threshold of 120 s for AFib verification compared to the 30 s used in the study (13). Two studies, similar with design and follow-up duration to ours, also showed lower detection rates-−29.2 and 23.6% at mean 17 and 12 months follow-up duration (9, 10). Mean age is comparable in Israel et al. and Victor et al. studies, while CRYSTAL AF has 7 years younger population, which can explain our higher detection rate considering age as a risk factor for AFib.

### ECG Biomarkers

PQ Interval (or PR interval) is included in the AFib risk score developed in the Atherosclerosis Risk in Communities study, with HR (for ms) 1.23 (19). Studies conducted by Cheng et al. and Magnani et al. included Framingham Study participants in determining long-term outcomes individually with prolonged PR(PQ) interval. The Cheng et al. study showed that each 20-ms increment in PR(PQ) was associated with an adjusted HR of 1.11 for AFib, like our study that showed an adjusted HR of 1.09. Similarly, when the PQ interval was grouped to the presence of atrioventricular block, the increased risk stayed comparable, i.e., two-fold as in the Cheng et al. study (12, 20). Smith et al. analyzed PQ interval components and concluded that P-wave onset to the P-wave peak that represents interatrial conduction (and surrogate of left atrial enlargement) is most strongly associated with AFib (21). Our results are comparable to the CRYSTAL-AF study since we also found age and PQ interval to be associated with AFib detection. However, we considered the CHAD2SVASC2 score to be more representable of disease burden and did the majority of analysis with it. Furthermore, the risk in our cohort was more pronouncedly dependent on the PQ interval (HR 1.96) in comparison to the CRYSTAL-AF study (HR 1.17 or 1.58). Although the prolonged PQ interval does not intuitively correlate with the development of AFib since it prolongs the conduction of the atrial impulses, it could be a sign of cardiac structural musculature changes that consequently lead to AFib. In one study on 70 patients, PTFV1 was found to be weakly associated with AFib after ESUS and ILR, however not with the recurrent ischemic strokes (22). The study did not report PQ interval measurements. The authors acknowledge the limitation of PTFV1 due to P-wave amplitude low voltage and mathematical computations necessary for value derivation. As suggested, the more advanced automated algorithms are needed to optimize the predictive values of PTFV1 and other ECG indices (22).

Supraventricular extrasystoles are significantly associated with AFib detection in our cohort. Thus, we could corroborate finding from Israel at al. (9). However, our study is not directly comparable since the authors recorded the occurrence of SVEs qualitatively, and we used the number of SVEs per hour. Additionally, the sensitivity of SVEs numbers is relatively low in our cohort, only 51%, while specificity is moderate-−77%. Cardiovascular health study, a prospective multicentre cohort, showed good AFib-risk discrimination value for SVEs per hour (23). The Copenhagen Holter Study recruited a healthy population, between 55 and 75 years of age with 6-year follow-up, supraventricular ectopic complexes (SVEC) with each increment of SVEC/hour as a continuous variable were associated with the occurrence of atrial fibrillation, in line with our observations (14).

### Timing of Detection

Similar to Israel et al., we detected more than half of our patients in the first months after implantation. However, Victor et al. reported a median of 30 days after implantation, probably reflecting a later time from event to implantation (60 days in Victor et al. study, 20 days in our and Israel et al. study). Time from the qualifying ESUS event to implantation could be responsible for different detection rates between studies.

### Oversensing

Although we do not have exact data on oversensing and false detection rates, we acknowledge that the significant reports send for adjudication fall in this category. The false-positive are responsible for up to 60% of scheduled transmissions and are due to pause (77%) and bradycardia (60%) (24). As noted by other authors, this poses a significant burden on practicing clinicians since one report adjudication lasts up to 30 mins. In the light of the broader utilization of ILR, ameliorated strategies should be implemented (24).

### CHA_2_DS_2_-VASc Score

Studies have shown that this score is also able to predict the AFib risk in patients in sinoatrial rhythm (10, 25). In our cohort, only two (1.9%) patients with CHA_2_DS_2_-VASc = 2 had had AFib, similar to the Israel et al. study where only one patient with a score of two had AF (9). In our cohort, the occurrence of AFib rises abruptly with a score > 3, with patients with a score of 5 having the highest risk for AF. However, it loses significance when adjusted to electrocardiographic parameters.

### Neuroimaging

Although univariate association for the presence of fragmented infarction was significantly associated with the AFib detection, this was not the case anymore in multivariable analysis. Previous reports showed ambiguous predictive values of infarct patterns for AFib detection. Territorial infarction in the middle or posterior cerebral artery seems like a plausible predictor candidate; however, we and others could not confirm the association (9, 26). The bilateral infarction could be associated with Afib detection after ILR implantation for ESUS, as shown in one recent study (26). The reasons for this ambiguity are not clear but could be explained by the intrinsically low AFib detection rate in ESUS, clearly suggesting other etiologies responsible for infarction patterns traditionally regarded as cardioembolic-appearing. Therefore, the patient selection for ILR should not weigh too extensively on neuroimaging characteristics, with the possible exception for the presence of bilateral infarction pattern.

### Clinical Importance

While AFib detection changes therapeutic decisions almost imminently, the causality of AFib to stroke is open to debate. Several studies were not conclusive to establish a direct link between AFib detection and the subsequent emerging stroke. Duration of AFib (for which we have not accounted for) is also debated; however, 120 s cut-off is widely accepted. Whether late-detected AFib (> 12 months of observation) are still causative for qualifying ESUS events is also debatable.

### Limitations

The limitations of the study are few but important. We have no data on the body surface index (BSA) and left atrial volume (LAV). Accordingly, we could not calculate LAD or LAV index values (LADI and LAVI). LADI is more readily measured than LAVI and will be used as one of the markers for atrial cardiopathy in ARCADIA prospective trial (27). LAVI reflects the extent of atrial cardiopathy more accurately, since the atrium size and shape vary between the patients (28), and was shown to be the good predictor for future AFib events after ESUS, where multivariable analysis showed a significant association with AFib detection in ESUS patients (28, 29). Furthermore, we have no values of laboratory biomarkers in each patient, so we could not draw firm conclusions from them. Inherent to automatic detection algorithm is the possibility that constantly detected tachycardias overrun storage capacity so that true AFib is not manually resolved. Furthermore, exact P wave characteristics such as P wave onset to peak, PTFV1, and P wave duration were not measured.

## Conclusion

In summary, we confirmed the feasibility of neurologist-led indication, implantation, and evaluation of ILR in the setting of embolic stroke of the undetermined source. The detection rate of AFib and previously reported prognostication parameters were replicated. The neurological management of complex ILR devices should be recommended.

## Data Availability Statement

The raw data supporting the conclusions of this article will be made available by the authors, without undue reservation.

## Ethics Statement

Ethical review and approval was not required for the study on human participants in accordance with the local legislation and institutional requirements. Written informed consent for participation was not required for this study in accordance with the national legislation and the institutional requirements.

## Author Contributions

CR, SP, and JM conceptualized the study. CR and UL were involved in the patient management and the detection of AFib. TZ, BG, and CH collected the data. SP analyzed data and drafted the manuscript. All authors reviewed and edited the manuscript and approved the final version of the manuscript.

## Conflict of Interest

The authors declare that the research was conducted in the absence of any commercial or financial relationships that could be construed as a potential conflict of interest.

## Publisher's Note

All claims expressed in this article are solely those of the authors and do not necessarily represent those of their affiliated organizations, or those of the publisher, the editors and the reviewers. Any product that may be evaluated in this article, or claim that may be made by its manufacturer, is not guaranteed or endorsed by the publisher.
