# Supplementary material for: Neurologist-Led Management of Implantable Loop-Recorders After Embolic Stroke of Undetermined Source
Source: Front Neurol. 2022 Jan 28;12:816511. doi: 10.3389/fneur.2021.816511 (PMC8831881; doi:10.3389/fneur.2021.816511)
Supplement: Supplementary file 1 [file Data_Sheet_1.pdf]

| Variables | β coefficient | HR (95% CI) | Wald test | P |
|---|---|---|---|---|
| **Age at implantation (years)** | 0.07 | 1.07 (1.03-1.11) | 13.87 | <0.001 |
| **$CHA_2DS_2$-VASc score** | 0.33 | 1.39 (1.12-1.72) | 8.94 | <0.001 |
| **Nr. of stroke/TIA in history** | 0.56 | 1.75 (1.20-2.54) | 8.55 | 0.003 |
| **Stroke/TIA in history** | 0.84 | 2.31 (1.28-4.17) | 7.74 | 0.005 |
| **Pre-mRS** | 0.25 | 1.29 (1.02-1.62) | 4.67 | 0.031 |
| **Arterial Hypertension** | 0.72 | 2.06 (0.92-4.61) | 3.06 | 0.080 |
| **Myocardial infarction** | 1.09 | 2.97 (1.06-8.30) | 4.29 | 0.038 |
| **Fazekas score (0-3)** | 0.25 | 1.28 (0.95-1.72) | 2.68 | 0.102 |
| **Left atrial enlargement** | 0.72 | 2.05 (1.12-3.72) | 5.50 | 0.019 |
| **Left ventricular function (normal)** | 4.68 | | | |
| **mild impairment** | 0.95 | 2.62 (1.10-6.22) | | 0.029 |
| **moderate impairment** | -15.03 | 0.00 (0.00-Inf) | | 0.996 |
| **Troponin -T (ng/ml) (log)** | 0.35 | 1.42 (1.10-1.82) | 7.37 | 0.007 |
| **pro-BNP (ng/ml) (log)** | 0.36 | 1.43 (1.18-1.75) | 12.81 | <0.001 |
| **PQ interval in ms** | 0.01 | 1.01 (1.01-1.02) | 15.53 | <0.001 |
| **PQ interval per 50 ms increase** | 0.60 | 1.82 (1.25-2.65) | 9.66 | 0.002 |
| **PQ interval >200 ms** | 0.94 | 2.55 (1.13-5.75) | 5.09 | 0.024 |
| **SVEs per hour (IHS)** | 0.25 | 1.29 (1.10-1.51) | 9.55 | 0.002 |
| **VEs per hour (IHS)** | 0.25 | 1.29 (1.11-1.49) | 11.73 | 0.001 |
| **Nr of SV runs (IHS)** | 0.25 | 1.28 (1.08-1.52) | 7.71 | 0.005 |
| **SV runs present (yes vs. no)** | 0.61 | 1.85 (1.02-3.36) | 4.04 | 0.044 |
| **VEs Couples (IHS)** | 0.24 | 1.27 (1.06-1.51) | 6.78 | 0.009 |
| **VEs Triplets (IHS)** | 0.67 | 1.95 (1.32-2.88) | 11.37 | 0.001 |
| **Any type of VEs present** | 0.92 | 2.52 (0.99-6.39) | 3.76 | 0.052 |
| **Event to implantation in days** | -0.00 | 1.00 (0.99-1.00) | 2.70 | 0.100 |

VEs – ventricular extrasystoles, SVEs – supraventricular extrasystoles, LDL-C – low-density lipoprotein cholesterol, log – log-transformed, IHS – inverse hyperbolic transformation

Supplemental Table I. Cox-proportional hazard univariate analysis of predictors for atrial fibrillation detection in 131 patients with embolic stroke of undetermined source.

Implantable loop recorder in real-world setting, association with detection of atrial fibrillation

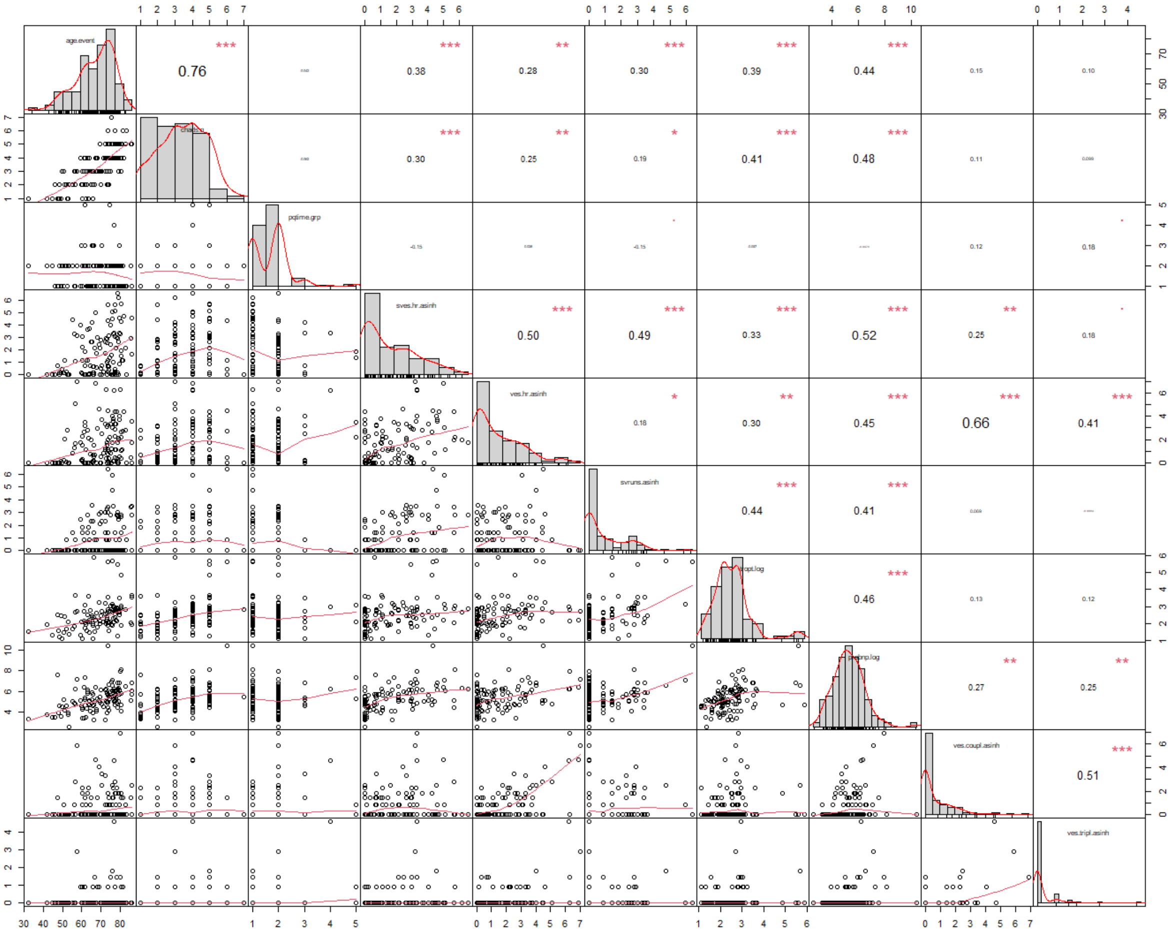


Supplemental Figure I. Correlation among parameters that were candidates for multivariable analysis, N=131 patients.

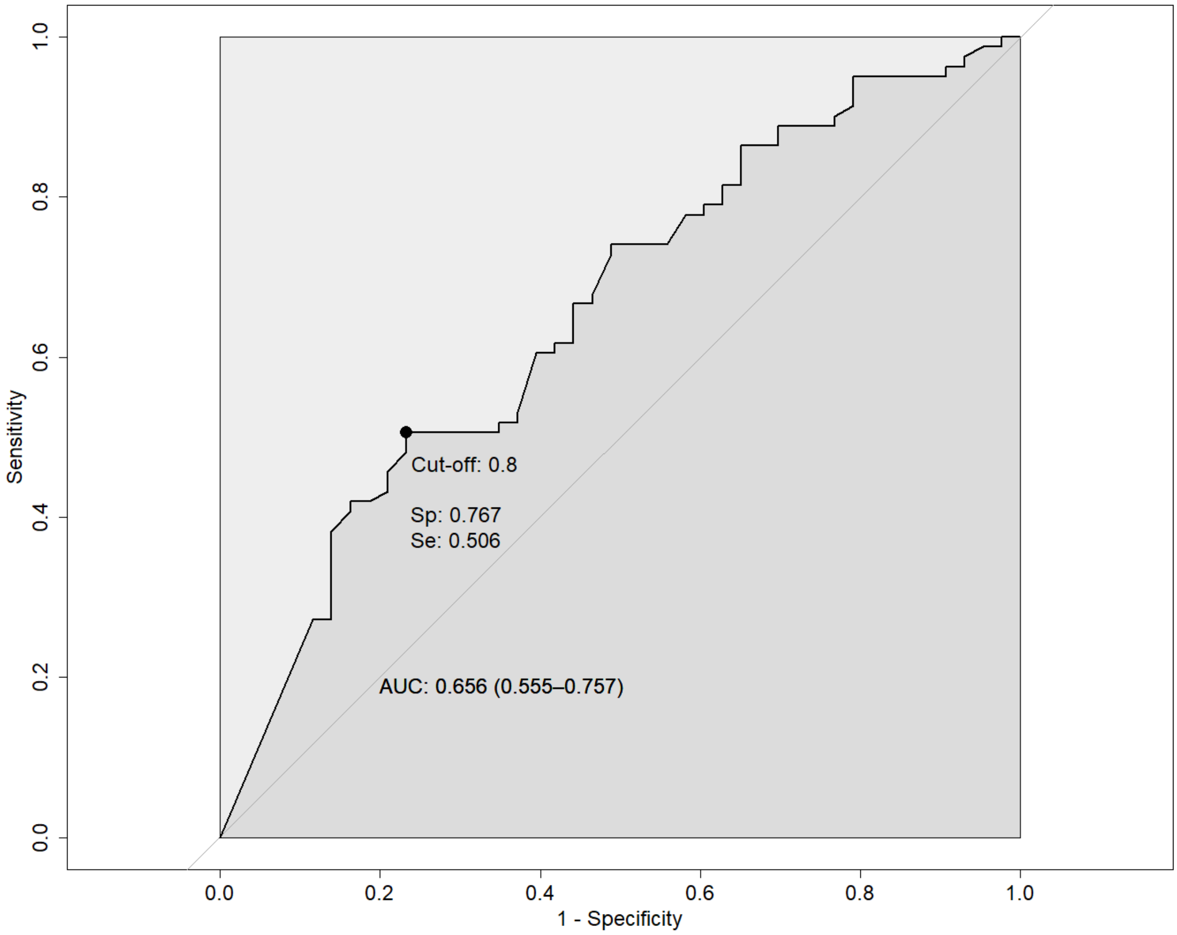


Supplemental Figure II. Receiver operating curve depicting detection performance of supraventricular extrasystole (as per hour, detected on holter monitoring prior to loop implantation) for atrial fibrillation detetction in 104 patients with ESUS.

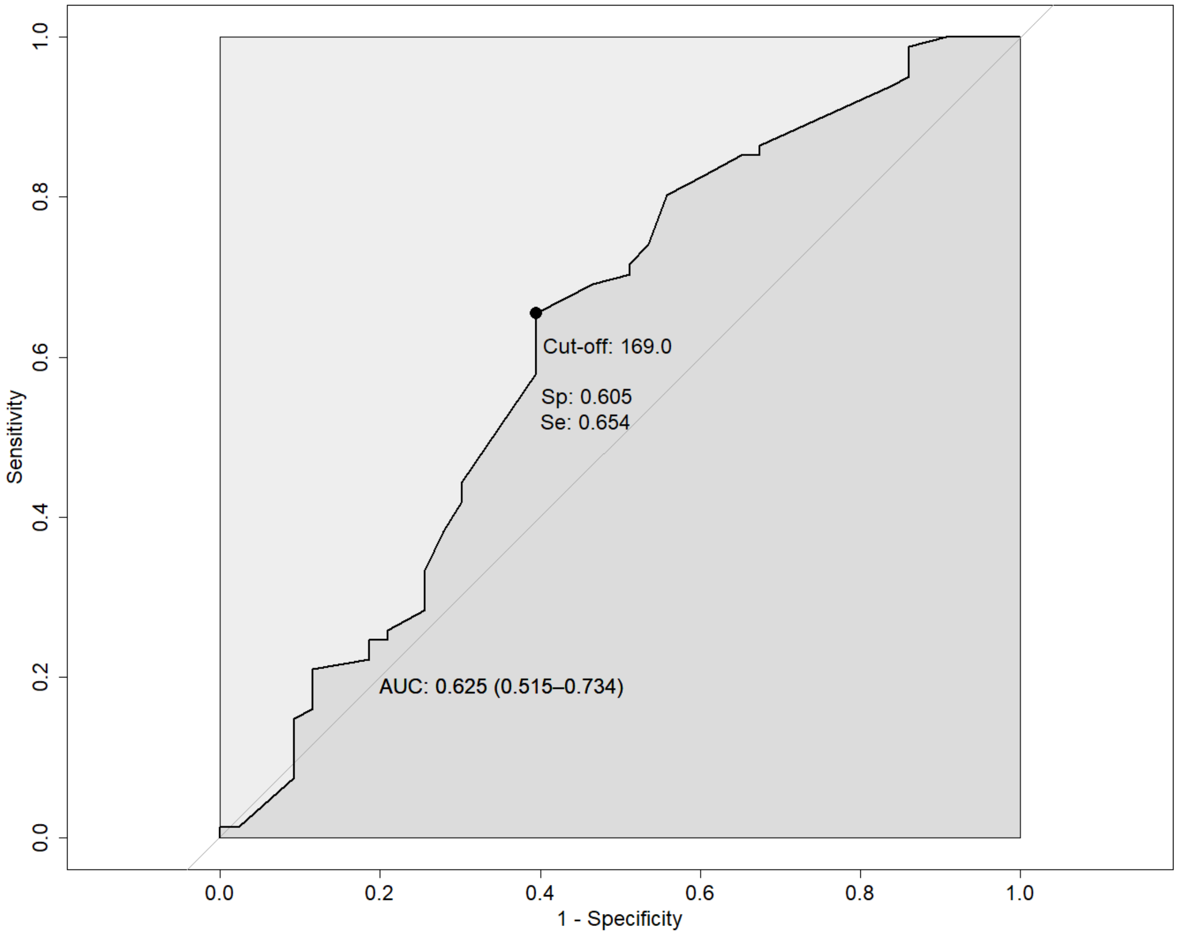


Supplemental Figure III. Receiver operating curve depicting detection performance of PQ interval (as millisecond, measured on admission EKG prior to loop implantation) for atrial fibrillation detetction in 104 patients with ESUS.

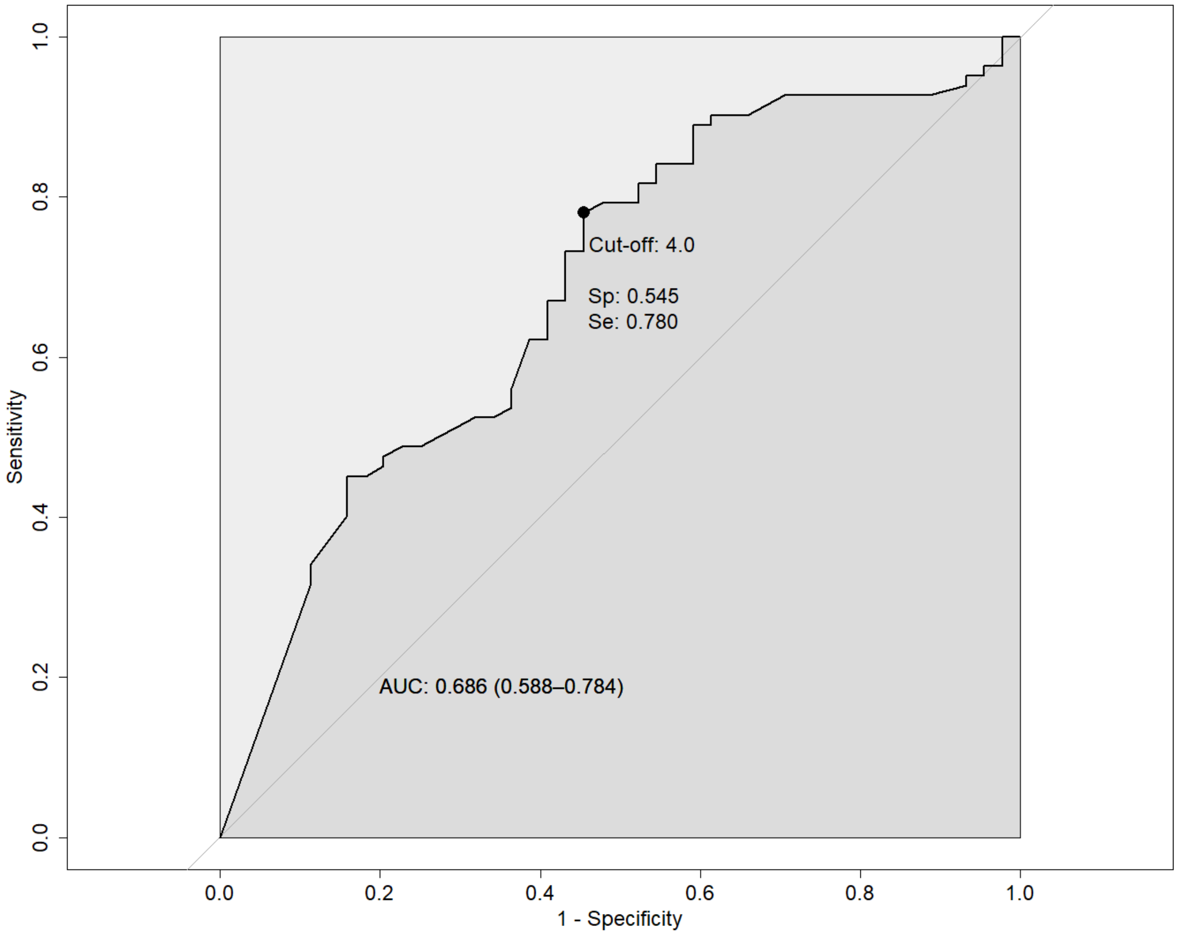


Supplemental Figure IV. Receiver operating curve depicting detection performance of ventricular extrasystole (as per hour, detected on holter monitoring prior to loop implantation) for atrial fibrillation detetction in 104 patients with ESUS.

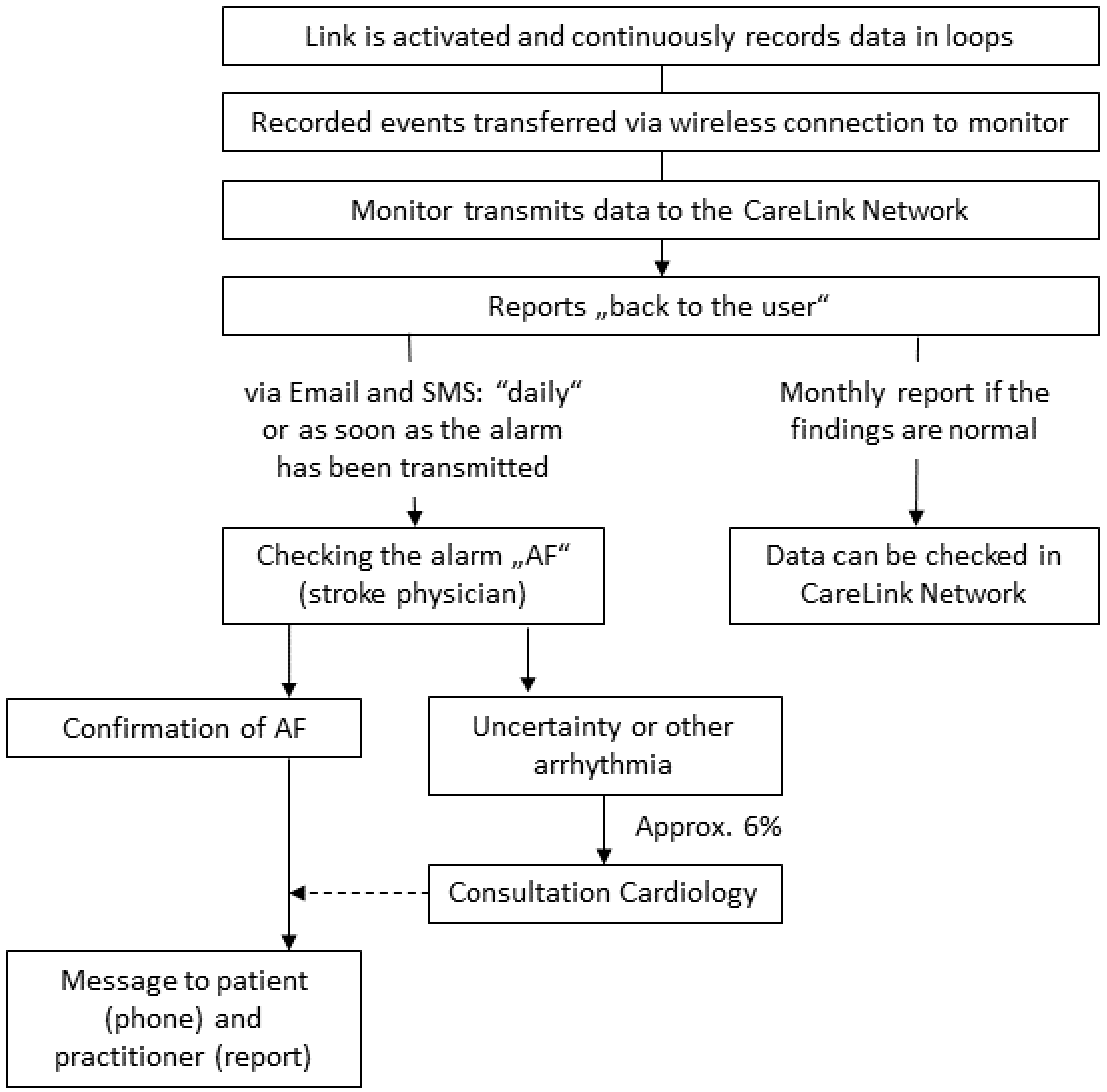


Supplemental Figure V. Review protocol for patients with implantable loop recorder at stroke unit, Christian-Doppler-Clinic, Salzburg, Austria.

AF – atrial fibrilation
